# Utilizing Midwifery-Led Care Units (MLCU) for Enhanced Maternal and Newborn Health in India: An Evidence-Based Review

**DOI:** 10.7759/cureus.43214

**Published:** 2023-08-09

**Authors:** Lily Podder, Geeta Bhardwaj, Alfisha Siddiqui, Rachna Agrawal, Ajay Halder, Manisha Rani

**Affiliations:** 1 Obstetric and Gynecological Nursing, All India Institute of Medical Sciences, Bhopal, Bhopal, IND; 2 Obstetrics and Gynecology, Santosh Hospital, New Delhi, IND; 3 Obstetrics and Gynecology, Sarojini Naidu Medical College, Agra, IND; 4 Obstetrics and Gynecology, All India Institute of Medical Sciences, Bhopal, Bhopal, IND; 5 Child Health Nursing, All India Institute of Medical Sciences, Bhopal, Bhopal, IND

**Keywords:** midwives, midwife led care unit, midwifery practice, newborn health, maternal health

## Abstract

The allocation of the midwife-led care unit (MLCU), a midwifery-led care model in which midwives carry out eminent roles to enrich maternal and newborn outcomes with minimal standard interventions, has appeared to be productive in furthering the quality of care and positive childbirth experiences. In the present article, we review the investments needed in MLCUs for their inclusion into the public health system by describing their advantages, the latest trends in maternal mortality, the roles of midwives, the relevant background, and the current advances in midwifery practices in India. Midwifery-led care is directed by a philosophy that considers pregnancy and childbirth as normal physiological events for women. Making use of a midwife, especially in low-risk pregnancies, extends satisfactory and cost-effective care. The Government of India has begun to introduce midwifery services to the country to improve the quality, righteousness, and worthiness in the provision of care and to offload higher-level hospitals. The year 2020 was designated as the "Year of the Nurse and the Midwife" by the WHO, highlighting the importance of nurses' and midwives' roles in sustaining quality health care. Further, the acceptability among clinicians and the public is crucial for the future advancement and implementation of MLCUs in India.

## Introduction and background

Advancing maternal-child health is pivotal to any nation's healthcare development [1]. However, despite nations worldwide devoting substantial resources to maternal-child health for years, maternal and child health problems remain an international health concern, notably in developing countries [2]. In the present article, we review the investments needed in midwife-led care units (MLCUs) for their inclusion into the public health system of India by describing their advantages, the latest trends in maternal mortality, the role of midwives, the relevant background, and the current advancements of midwifery practices in India.

Severe maternal outcomes: globally

The maternal mortality ratio (MMR) remains high across the world. Approximately 295,000 women died from complications related to pregnancy and childbirth in 2017. Most of these (94%) deaths occurred in low-resource settings. Sub-Saharan Africa and Southern Asia reported approximately 254,000 (86%) of the figured global maternal deaths. Sub-Saharan Africa singly reported about two-thirds of the maternal deaths, whereas Southern Asia reported about one-fifth. These high MMRs in some countries accentuate the wide gap between the MMR of developed and developing countries [3]. The MMR in developing countries in 2017 was 462 per 100,000 live births, compared to 11 per 100,000 live births in developed countries [4].

Severe maternal outcomes: India

India shares 15% of global maternal deaths [5]. In accordance with the newest reports of the National Sample Registration System, India's MMR decreased from 130 per 100,000 live births from 2014 to 2016 to 113 per 100,000 live births from 2016 to 2018. The overall number of annual maternal deaths in India decreased from 33,800 in 2016 to 26,437 in 2018. Complications that account for more than two-thirds of all maternal deaths include postpartum hemorrhage, puerperal sepsis, hypertensive disorders, and unsafe abortions [6]. India must achieve the MMR target set in its millennium development goals, and it faces a mammoth challenge to reach its sustainable development goals (SDG) target of an MMR of under 70 per 100,000 live births by 2030 [7]. Moreover, recent evidence has drawn attention to the need for more respectful maternal and child healthcare, given that many women endure abuse, negligence, and other mistreatment during childbirth experiences [8].

Midwife-led care units

Evidence shows that midwifery-led maternal health services were efficacious as an adjunct in reducing severely negative maternal outcomes. Midwife-led care includes comprehensive obstetric and newborn care provided by a midwife either at an in-site hospital-based care unit or as a stand-alone care unit outside the institutional referral center. In-hospital care units have proven to be beneficial in improving poor access to maternal health care and difficult referral systems [9]. The provision of midwife-led continuity of care by competent midwives has been shown to decrease neonatal deaths by 16% and preterm births by 24% [10]. Midwives provide care related not only to health outcomes that reduce mortality and morbidity but also to patients' overall health and well-being, ensuring mother-baby bonding and family participation. Midwife-led models of care can mitigate interpersonal issues by promoting quality care, emphasizing prevention, support, and deferential relationships, and fostering women-centric childbirth care [11]. The Lancet Series on Midwifery (2014) estimates that, when educated and governed according to international standards, midwives can dispense 87% of the necessary care to mothers and newborns, including maternal care, newborn care, breastfeeding, family planning, and screenings for HIV infection, tuberculosis, and malaria. This model of care is well substantiated worldwide [12]. Enhancing maternal health services by establishing quality midwifery-led services will improve locally accessible quality care for all childbirths and remarkably decrease maternal deaths. Midwives must be reasonably assigned, be approachable by the population, and possess the necessary capabilities to deliver relevant and satisfactory quality care. Midwifery in India has yet to be approved as a separate profession, both technically and by society [13]. In 2018, the Ministry of Health and Family Welfare (MoHFW) published guidelines on midwifery services in India and provided direction for the education, training, and quality assurance of midwifery services in India, along with methods by which to incorporate this model of care into the existing public health system to accomplish SDGs [14]. Hence, as an alternative model of delivery of care, the midwifery-led care model commands consideration, especially in a country such as India, which has a wide disparity in healthcare availability based on class, geographic, and cultural barriers [15].

## Review

Search strategy

We executed an extensive literature review by browsing several databases, namely PubMed, Scopus, Web of Science, Google Scholar, several obstetrics and gynecology organizations, associations' web pages, and the Government of India Guidelines, using the following keywords and phrases: "maternal health," "newborn health," "midwifery practice," "nurse practitioner in midwifery," "midwives," "severe maternal outcome," and "midwife-led care unit." We also screened the reference list of selected articles to examine other relevant articles. We subsequently assessed the selected literature and synthesized the findings to include them in the present review article under the following subthemes: severe maternal outcomes in India and globally, the role of midwives in maternal and newborn health, midwifery practices in India and globally, and the future of midwifery in India.

Literature review

Compared to other countries, India's healthcare system offers little independent midwifery-led care for women during pregnancy, childbirth, and the puerperium period [4]. In the present review, we explore the effectiveness of midwifery-led care to determine that an alternative model of care delivery (i.e., MLCUs) is required to improve maternal and newborn survival, reduce maternal and neonatal mortality and morbidities, control medicalization during pregnancy and childbirth, and secure the human dignity of childbearing women in India. We present this review to promote the integration and strengthening of midwifery services into India's existing public health system, as have several other countries, such as the United Kingdom, Sweden, and Australia [7,8].

MLCU components

Midwifery-led care is an evidence-based practice that involves improved maternal care. Ideal MLCUs involve midwives adequately skilled to aid childbearing women, providing care that promotes normal physiological pregnancy and childbirth and encourages and defends non-intervention during vaginal delivery [16]. Midwives exercising comprehensive care will abide by the values of respect, empathy, and human rights advocacy, ensuring the execution of competent and evidence-based practice to improve the quality of midwife care. The use of midwives promotes women-involved and informed decision-making as well as cooperation and consultation with other healthcare workers to comprehensively care for women, newborns, families, and communities. Midwives make use of resources appropriately and make timely referrals when complications arise. Table 1 presents the standards of optimal, philosophy-based care in this context by the International Confederation of Midwives (ICM) [17].

**Table 1:** Elements of midwifery care. Source: Table has been prepared by the authors of this review.

S. No.	Elements
1	Health education [18]
2	Family planning counselling and services [18]
3	Early identification and referral of complications [19]
4	Comprehensive preconception and antenatal care for adolescent girls and women [19]
5	Comprehensive intrapartum care with positive childbirth experience including basic emergency obstetric and newborn care (BEmONC) and timely referral for comprehensive emergency obstetric and newborn care (CEmONC) [18,19]
6	Comprehensive postpartum and postnatal care (breastfeeding and family planning education and provision of contraceptive services) [18]
7	Comprehensive postnatal care for all newborns including all elements of essential newborn care (ENC) [18]
8	Comprehensive abortion services based on dignified care and co-decision procedure [18,19]
9	Advocacy, leadership, and management that impart conduciveness to creation and continuation of propitious work environment [18]

Role of midwives in promoting maternal health and reducing adverse outcomes

The capacity of midwives to achieve SDGs has expanded over the past few decades. Midwifery lies at the core of health systems intended to improve maternal and child health, and efforts to improve such systems will be invigorated by strengthening midwifery care. Midwives ensure that women, newborns, and families receive adequate and timely care [18-20]. The ICM defines a midwife as 'A person who has successfully completed a midwifery education programme that is based on the ICM Essential Competencies for Midwifery Practice and the framework of the ICM Global Standards for Midwifery Education, and is recognized in the country where it is located; who has acquired the requisite qualifications to be registered and/or legally licensed to practice midwifery; and who uses the title "midwife" and demonstrates competency in the practice of midwifery' [21].

The Lancet Series on Midwifery revealed that implementing full-scope midwifery can improve over 50 aftereffects of childbirth for women, newborns, and families by mitigating risk, improving emotional and mental health, and preserving resources. Table 2 presents detailed data on the outcomes of MLCUs. Midwives can play a crucial role in advancing women's and babies' health from preconception through pregnancy and babies' infancy. However, notable social, political, and economic barriers have hindered the widespread availability of high-quality midwifery services [22]. Instead, midwives have provided evidence-focused, human rights-focused, high-quality, culturally conscious, and respectful services under the umbrella of the following elements [23].

**Table 2:** Maternal and neonatal outcomes with MLCUs. Source: Created by the authors of this review. MLCU: Midwifery-led care unit.

S. No.	Outcome indicators
1	Comprehensive maternal and child health care
2	Efficacy in lessening maternal mortality and morbidity
3	Decline in preterm birth and neonatal death
4	Mitigation of interpersonal issues involved in childbirth
5	Promotion of quality care
6	Accessible and affordable skilled care
7	Client satisfaction
8	Timely implementation of emergency measures
9	Cost effective and sustainable care measure
10	Efficacy in reducing Cesarean section and several medical interventions
11	Promoting psychosocial aspects of care
12	Augmentation of positive feelings of childbirth
13	Promotion of breastfeeding

MLCUs' utilitarian approach

MLCUs satisfy clients using a multipronged strategy [11]. The presence of midwives during labor satisfies patient expectations during childbirth regarding caretaker competencies and overall cost [24]. MLCUs also aid patients via counseling, nutrition guidance, hygiene training, and collaboration with obstetricians in high-risk cases. Further, MLCUs are equipped to implement emergency and family-planning measures if needed. Moreover, rates of hospitalization during the antenatal period and postnatal depression can be easily attenuated by the MLCU-centric approach [24, 25]. In a Swedish questionnaire-based study involving 2,686 participants, only 26% of women reported dissatisfaction related to the time and support provided by midwives [26]. Similarly, a randomized questionnaire-based comparative model (n = 1,000) highlighted satisfaction with midwifery care. The recently published Norway Trial also favored midwife-led birth care compared to obstetrician-led care in primary care settings [27].

Although MLCUs are cost-effective and sustainable for low-risk populations, their efficacy is questionable for high-risk cases. Evidence supports a higher usage of non-pharmacological pain relief methods in the context of MLCUs rather than instrumentation to promote patient satisfaction [28]. A systematic review targeted to analyze the cost-effectiveness of MLCUs concluded that the widespread usage of midwifery may help increase healthcare affordability for low- and middle-income populations as compared to consultant-led care [29]. A tertiary-care center-based retrospective cohort registry implemented in Lithuania included a total of 1,384 and 1,283 low-risk delivering women in 2012 and 2014, respectively; the study concluded that midwifery-led care proved efficacious in reducing cesarean procedures and several other medical interventions with no apparent increase in adverse neonatal outcomes [30].

Many childbearing women seek freedom of selection for their desired birthing place or method. To make such a decision, clients should be provided with all relevant information and requisite knowledge of available choices and a comprehensive explanation of the advantages and disadvantages of each. Demographic patterns, geography, risks, and availability influence a client's decision [31]. Although consultant-led care may be safer in terms of mortality rates, consultant-led care lacks the necessary psychosocial support.

Further, the presence of someone who is both empathic and skilled during the process of childbearing has benefits. Women may feel secure and emotionally comfortable in the presence of a midwife [18,19]. Midwives are trained to use their own physical and emotional energy to augment positive feelings according to patients' needs [32].

MLCUs must employ competent midwives equipped with the requisite skill sets and knowledge to provide optimal care. To supplement this, frequent obstetrician-led education programs and workshops aimed at conducting safe vaginal deliveries and newborn care should be organized. Additionally, midwives should be able to identify impending danger early and make timely referrals to higher centers [33]. The authors of one study observed that women who had access to midwife care sustained less pregnancy loss because of better antenatal care [34]. MLCUs not only increase the rate of normal vaginal deliveries and early breastfeeding initiation but also decrease the usage of invasive interventions such as anesthesia. A North America-based quantitative prospective study found a decreased occurrence of intrapartum and neonatal mortality in home-based deliveries conducted by skilled midwives [35]. Further, the University of California, San Diego, Women's Health and Reproductive Justice program found midwife participation to be beneficial in low-intervention, risk-free care, with data suggesting a 10% cesarean delivery rate and a 98% breastfeeding rate [36].

**Figure 1:**
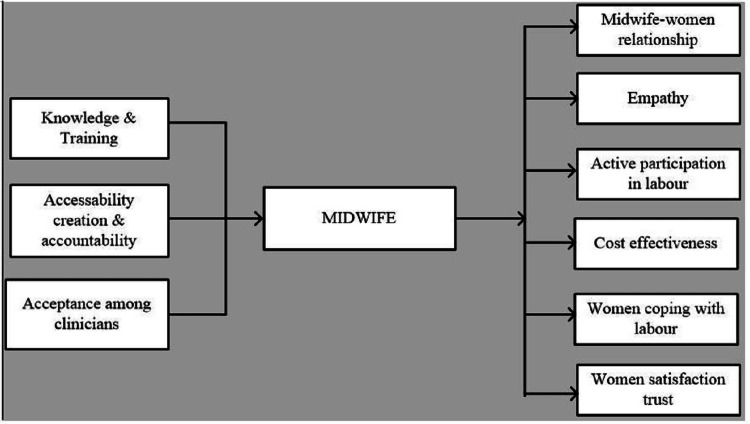
Schematic spectrum of midwife health services. Source: Created by the authors of this review.

Improving patients' confidence and feelings of self-efficacy during childbirth and newborn care consistently minimizes the associated negative impact and augments mood elevation and overall satisfaction. A US-based correlational descriptive study found that women using a midwife during childbirth possessed a greater level of personal control over and satisfaction with their experience [37]. There is a significant inverse relationship between maternal anxiety and personal control during the delivery process; MLCUs foster such maternal control by providing patient-centric care and involving women in formulating their birth plans. An ethnographic England-based study compared the roles of midwives in six areas (i.e., presence, relationships, stress coping, labor progress, birthing partners, and support) and found that individualized midwifery-led care during labor increases clients' overall satisfaction (Figure 1) [38].

Midwifery practices globally

Midwives have historically contributed to dwindling MMRs. Since the 1600s, Sweden has been the foremost innovator among Nordic countries in midwifery education. Sweden's land mass is extensive compared to its relatively small population, making the availability of doctors in rural areas a challenge. Midwifery as an educated and paid profession was developed and expanded throughout the country [39]. Currently, the Swedish public health strategy includes granting midwives an integral role in maternal care and developing public health policies, which has resulted in an expeditious reduction in MMR [40]. Presently, Sweden has the lowest MMR in the world. In the United States, the issue of the MMR has been deliberately undermined, and midwives have not been adequately integrated into the public healthcare system [38]. Currently, midwives attend just 8% of births, compared to almost two-thirds of births in the United Kingdom and the Netherlands. Malaysia and Sri Lanka have, over the past half of a century, reduced MMR from 500 to under 50 every seven to 10 years by posting clinically trained midwives throughout communities [41]. Four other developing countries, such as Bolivia, Yunan in China, Egypt, and Jamaica, halved their MMRs from 200 to 300 to 100 to 150 in less than 10 years by investing in maternal health care [40]. Overall, existing evidence suggests that access to skilled birth attendance is crucial to reducing MMRs.

Midwifery practices in India

Several studies have highlighted the origin of midwifery in India, which dates to British control, and the challenges in nursing and midwifery education, regulation, organization, and policy-making since. Presently, India's healthcare system necessitates competent, independent midwives [42]. In 2005, the National Health Mission, launched by the Government of India, put forth measures to compose operational guidelines to enforce midwifery education [43]. Table 3 highlights the challenges related to midwifery practice in India.

Rajasthan is the first Indian state that has sought to organize and regulate nurse practitioners (NPs). Although there are many NPs in the region, particularly in rural areas, their recognition by the population has not yet been legitimatized. In 2002, the state of West Bengal pioneered the adoption of a nurse practitioner in midwifery (NPM) course under the India-Aus AID project; this program provided 18 months of training for diploma and graduate nurses, sanctioned those nurses at 12 posts around their state and afforded them their due recognition in 2010 [43]. Similarly, in 2009, the government of Gujarat attempted to employ specialized midwives with competent skills in maternal and newborn care units, sanctioning 25 IMP posts, which unfortunately remained unfilled because of administrative challenges [42-43]. In 2011, Fernandez Hospital in Telangana implemented the Professional Midwifery Education and Training program, beginning a triadic connection between the state government, UNICEF, and Fernandez Hospital to train nursing students in midwifery through the NPM course and sanction 125 posts. From August 2011 to June 2017, midwives helped 6,317 birthing women [43-44]. In 2016, Choithram Hospital, a private institute in Madhya Pradesh, began NPM training for nurses without involvement from the state government [43]. Other states, including Kerala, Andhra Pradesh, and Tamil Nadu, took similar steps to train registered nurses in NPM, although participants could not be stationed in these areas because of regulatory hurdles [44].

Moreover, the Government of India developed a constructed role for registered nurse practitioners (RNPs) as community health officers (CHOs), which could be achieved after participating in a six-month training program. These CHOs are scheduled to provide healthcare services, leadership, supervision, and management and play a dynamic role in India's healthcare system [45].

Various midwifery-led care models have been introduced in India to explore variations in childbirth experiences, such as RN training programs, ICM standard compliance, and childbirth outcomes, in contrast to physician-led care models [41]. These include the Aastrika Birthing Center in Bangalore [46], the Sanctum Natural Birth Center in Hyderabad [47], Birth Village in Kochi [48], SWA-Choithram's Natural Birthing Center in Indore [49], and the midwife-led antenatal clinic in Vellore [50]. These projects are successful in increasing the visibility of midwifery. However, appropriate strategies are not in place for the stable and upscaled systemic integration of midwifery into the public health system to occur. This integration can be made possible with diligent planning and implementation through robust legislation, accreditation, careers, and scope of practice for NPMs (Table 3).

**Table 3:** Challenges of midwifery practice in India. Source: Created by the authors of this review.

Sl. no	Challenges	Source/s
1	Administrative hurdles	[40,43]
2	Regulatory failure/lack of regulatory framework	[13,40]
3	Inappropriate strategies for implementation	[4,39]
4	Deficient clarity regarding Midwife’s Role	[5,43]
5	No suitable plan for career progression pathway	[13]
6	Deficit in amendment of imperative act	[13]

Effective implementation of midwifery practices in India

Role of NPMs and Integration Into the Existing Healthcare System

**Figure 2:**
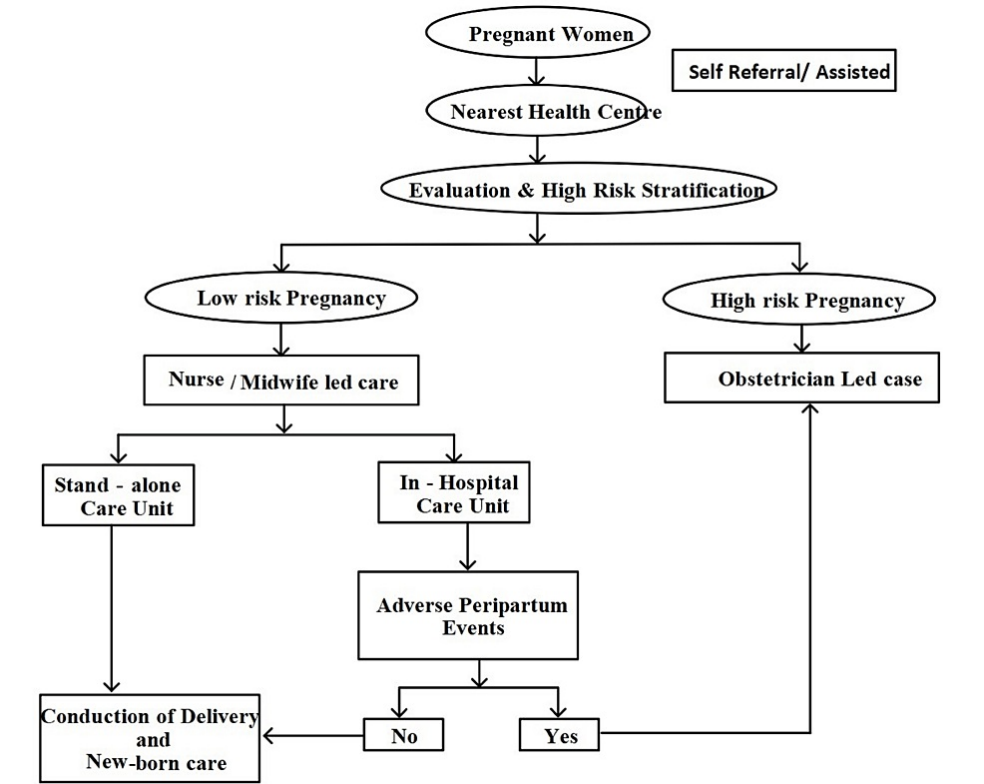
Importance of MLCU in maternal healthcare and referral algorithm. Source: Created by the authors of this review. MLCU: Midwifery-led care unit.

The recognition of midwives, auxiliary nurse midwives, and staff nurses is necessary to generate evidence-based policies [39]. There is a lack of clarity regarding the roles of midwives within health systems, and their limited scope of practice has led to significant variations in how midwifery is integrated into health systems [40]. The successful execution of midwifery programs in any state will chiefly be determined by the induction of midwives into the existing healthcare system [7]. Considering the need for trained human resources to provide quality care to 30 million pregnancy cases every year in India, and recognizing the existing challenges at the same time, an alternative model of service provision is essential for strengthening reproductive, maternal, and neonatal health services in India [9]. Midwifery care in India can further serve as a cost-effective, efficient model to provide quality care and reduce over-medicalization, guided by the Ministry of Health and Family Welfare for midwifery care in India [39,42]. Midwifery-led care encourages task shifting from doctors to midwives in relation to the promotion and conduction of physiologically normal births and reduces unnecessary interventions, including cesarean sections. In this system, pregnant women who experience complications will be referred to a specialist in the First Referral Unit or the Special Newborn Care Unit, which should be accessible immediately (Figure 2) [39-40].

NPM Career Progression

A career progression pathway for NPMs has not been suitably planned after introducing midwives into the existing healthcare system, making it challenging for those newly appointed to proceed as members of an autonomous profession. Moreover, the midwifery profession is not yet regulated and is consequently proscribed to work as a separate cadre in the health system. Thus, even after completing midwifery training, midwives are often not allowed to work within their scope of practice and competence. It is of key importance to deploy midwives in their determined professional areas so they can engage in their full scope of practice and promote positive health outcomes for women, newborns, and families [51].

Strengthening of Legal and Regulatory Framework

A lack of regulatory framework is a primary factor negatively affecting midwifery in India. The Indian healthcare system confronts various hurdles in healthcare provisions, including poor standards, unspecific regulatory functions, and insufficient amends. These deficits have hindered the creation of leadership posts, thereby debilitating the progress of the midwifery profession. Many studies have evidenced the significance of midwifery for maternal and neonatal health, indicating the necessity of making lawful attempts to legitimize NPMs as an autonomous profession independent of generalized nursing [51].

Midwife Training

The National Midwifery Task Force, led by various experts, revealed that further post-basic education (i.e., 18 months of training in theory and clinical experience along with competency-determined training sessions) will be required to harness competent midwives. Additionally, the Indian Nursing Council stated that a range of innovative educational strategies can be practiced in both academic and clinical settings to foster learning experiences [52]. It is integral to acknowledge and address the flaws of India's midwifery regulation, practice, and education, particularly given that 83% of maternal deaths, stillbirths, and neonatal deaths may be prevented when NPMs lead care.

The suggested solution to address the challenges (Author's own creation based on reviews) includes the establishment of a separate Directorate for Midwifery Education and Practice; the establishment of a separate Midwifery Council at both Central and state levels to regulate Midwifery Practice in India; the framing of a Midwifery Bill to standardize Midwifery Practices throughout the nation; the formulation of job specifications and descriptions for Nurse Midwives at all levels of care after adequate brainstorming; the building of supervision mechanisms across the healthcare system; the provision of adequate investment for Midwifery education and practice in India; the legitimization of NPMs as an autonomous and independent profession; networking and collaboration with Central and State government agencies and local administrative authorities to implement and facilitate midwifery practice; ensuring that trainee midwives are adequately trained by maintaining student and case load ratios, with the adoption of innovative strategies in academic and clinical settings to foster their learning experiences; and the timely review of the scope of midwifery practices.

## Conclusions

There is adequate evidence to support the beneficial aspects of midwifery care in promoting maternal and newborn health. Governmental leadership in enhancing midwife education, employment, and effective utilization in health centers is crucial to improving maternal and newborn care in India. Respectful treatment for clients and collaboration among obstetricians are other vital factors in sustaining high-quality midwifery practices. Additionally, clinicians and researchers should strengthen MLCU participation through evidence-based research. Moreover, cooperative interdisciplinary training and the development of effective skill sets among midwives can be promoted by involving experts in the fields of obstetrics and gynecology, pediatrics, and public health.
